# Digitizing Scoring Systems With Extended Online Feedback: A Novel Approach to Interactive Teaching and Learning in Formative OSCE

**DOI:** 10.3389/fmed.2021.762810

**Published:** 2022-01-25

**Authors:** Chia-Chen Wang, Yu-Chin Lily Wang, Yu-Han Hsu, Haw-Chyuan Lee, Yu-Chan Kang, Lynn Valerie Monrouxe, Shao-Ju Chien, Te-Chuan Chen

**Affiliations:** ^1^Department of Pharmacy, Kaohsiung Chang Gung Memorial Hospital, Kaohsiung, Taiwan; ^2^Faculty of Medicine and Health, University of Sydney, Sydney, NSW, Australia; ^3^Division of Pediatric Cardiology, Department of Pediatrics, Kaohsiung Chang Gung Memorial Hospital, Kaohsiung, Taiwan; ^4^School of Traditional Chinese Medicine, Chang Gung University College of Medicine, Taoyuan, Taiwan; ^5^Division of Nephrology, Department of Internal Medicine, Kaohsiung Chang Gung Memorial Hospital, Kaohsiung, Taiwan; ^6^School of Medicine, Chang Gung University College of Medicine, Taoyuan, Taiwan; ^7^Chang Gung Memorial Hospital Linkou Branch, Chang Gung Medical Education Research Centre, Taoyuan, Taiwan

**Keywords:** OSCE, interactive, online, feedback, digitizing scoring

## Abstract

**Objectives:**

Objective structured clinical examinations (OSCEs) are common for formative assessment. We developed an Online Smart Communicative Education System and aimed to explore the factors that affect the perceptions of both teachers and students for teaching and learning.

**Methods and Analysis:**

A two-year cross-sectional cohort study was undertaken. The program includes three parts. Part I Pre-OSCE: an online flipped class in preparation for task-related knowledge and skills. Part II OSCE-day: 10 tasks in one track formative OSCE. Part III Post-OSCE: extended online feedback for participants with further questions after the exam and raters with more feedback after reviewing their performance online. Principal component analysis with varimax rotation was performed to analyze the perceptions of students and teachers to the Online System by means of questionnaires.

**Results:**

Seventy-six pharmacy students (male 32.9%) took the exam and 24 raters (male, 25%) participated in the scoring during the OSCEs. The mean G coefficient was 0.88. Seventy-six questionnaires from the students were obtained for the analysis. Results explained the cumulative variance of 73.9% for *component (1) “Effects of extended online feedback”*: 40% and (*2) “Facilitation of learning”*: 33.9%. Thirty-nine questionnaires from the raters who experienced the Online System were obtained for the analysis (male 23.1%). Results explained a cumulative variance of 77.3% for *component (1) “Effects of extended online feedback”*: 36.6%, (*2) “Facilitation of scoring and feedback”*: 24.5%, and (*3) “Feasibility of online platform”*: 16.2%, respectively.

**Conclusion:**

We demonstrated good reliability for digitizing the scoring system with educational support to facilitate teaching. “Effects of extended online feedback” was the major aspect in explaining the variance from the perceptions of students and raters by factor analysis. In comparison with traditional formative OSCEs, extended online feedback is a novel approach, which extends the process of learning and teaching among the learners and raters and overcomes the barriers of time limitation and distance.

## Introduction

The Objective Structured Clinical Examination (OSCE) is an evaluation tool commonly adopted in healthcare professions education to assess the core competencies of healthcare students or professionals (1). The OSCE uses direct observational techniques and comprises numerous “stations” focusing on a variety of skills and behaviors. Assessment can be formative or summative. The former provides the benchmarks to orient learners who are approaching an unstructured body of knowledge, guiding their future learning, providing reassurance, promoting reflection, and shaping values (2). However, OSCEs are not without challenges. For example, they are expensive, time consuming, and require manpower. Additionally, mistakes can be made due to illegible handwriting, and intensive examiner training is required (3). Further, adequate space for construction is also required (4) alongside significant resource support. It also takes time for traffic transportation to gather the students and raters in one, or several, OSCE sites, especially when they are distributed across multiple campuses and hospitals. However, they are good assessments, either formative or summative, when evaluating competency-based medicine around integrative knowledge, skills, and attitudes due to their well-designed blueprint.

The application of new technology is growing and changing how we teach, learn, and practice clinically (5, 6). This includes various mobile and hand-held electronic devices (7). The rating of students' performance in an OSCE setting can now be effectively and efficiently recorded in a digital form (8, 9). Individual students' data can be immediately accessed for one-to-one feedback, and all these data can be synchronized to provide immediate, automated, highly elaborated, and formative feedback for the whole group at the end of the OSCE. The reliability and validity of the assessment process and the potential for application in nursing and midwifery education had been reported (10). However, this research failed to examine the effects on teaching and learning processes.

Effective feedback is crucial for the learners to build up their knowledge, skills, and attitudes to maximize their potential and professional development, to raise their awareness, and to identify adequate actions to improve their performance (11). However, learners complain about the lack of feedback they receive (12). Indeed, the research analyzing verbal interaction of feedback dialogue reported it as being skewed, predominantly toward positive or neutral aspects, and being overly centered on the teacher's role, underemphasizing that of the learner (13). Furthermore, an adequate feedback provision is difficult in a traditional formative OSCE setting. Finally, research investigating students' memory of feedback during OSCEs found that residents could recall very few feedback points immediately after the OSCE, and a month later. In addition, the feedback points that had been recalled were neither very accurate nor representative of the actually provided feedback (14).

The use of OSCEs in pharmacy education is a growing trend, particularly with the greater demands for accreditation requirements (15–17). Indeed, it is the unbiased and objective elements (due to multiple assessors) that have led OSCEs to become “a mandatory and critical norm” for evaluating core competencies (17). The OSCEs may provide a broad assessment of competencies inclusive of patient counseling, interpersonal communication, clinical pharmacokinetics, identification and resolution of drug-related problems, and literature evaluation/drug information (17) in comparison with traditional written or oral tests. However, while the advantages of the OSCE are frequently cited, including having multiple examiners and stations that emulate real-world settings alongside reproducibility and acceptability, the issue of student feedback is frequently neglected (16, 18, 19). For example, in a systematic review of pharmacy articles focusing on teaching and learning *via* OSCE (2003–2016), in the description of all 14 eligible studies, the word “feedback” was never mentioned (16). Furthermore, in another review of the literature (2000–2015), only eight of the identified 42 articles (35%) reported on the issue of student feedback, with five studies reporting that no feedback at all was provided (18). In a traditional OSCE, students are provided with individual feedback following each task, with group debriefing occurring at the end of the examination covering common areas of concern. Each rater is only aware of the performance of the students at their own station. Thus, group debriefing is useful in informing both raters and students of common issues to facilitate their teaching and learning. Also, while every student typically receives written and individual feedback, there is limited time for teachers to give effective and critical feedback and for students to ask questions for clarity.

In summary, the challenge is for raters to give immediate individual feedback based on their observations of students' performance with written remarks; common mistakes are difficult for the director to immediately debrief post-exam without real-time information. We aim to develop a system to facilitate the scoring and feedback of the raters to ensure the learning of the students. Specifically, we aim (i) to test the effect of a digital scoring system on teaching; (ii) to explore the factors that affect the extended online feedback; and (iii) to test the facilitation effect on learning. The main research question is: What are raters' perceptions of teaching when using the digitizing scoring system, and those of the students on learning with extended online feedback in an OSCE setting?

## Methods and Analysis

### Context

The pharmacy education system in Taiwan is mainly divided into a 4-year program with a Bachelor of Science and a 6-year program with a doctor of pharmacy. Pharmacy students may apply for a 2-year post-graduate (PGY) program for clinical training based on patient-centered holistic care. This study is conducted in 14 hospitals in four areas in the south of Taiwan, where they joined the same pharmaceutical clinical skills training program. The formative OSCEs comprise a ten-station track with 2-min immediate feedback in each station and a group debriefing at the end of OSCEs. The scores of items were easily (unintentionally) missed, with errors of typing and calculation by the raters when using the traditional paper-based system. Additionally, students responded that they usually have further questions after the traditional OSCEs, despite their immediate feedback.

### Study Design

A 2-year cross-sectional cohort study was undertaken, which the Institutional Review Board Ethical Committee of Chang Gung Medical Foundation has approved. Fourth-year undergraduate year (UGY) and first-year PGY pharmacy students from 14 hospitals, alongside OSCE raters, were invited to participate in the study during the period of 2016–2017. The raters who had passed a training program for the raters were also recruited from the joint hospitals.

### The Online Smart Communicative Education System

**Figure 1:**
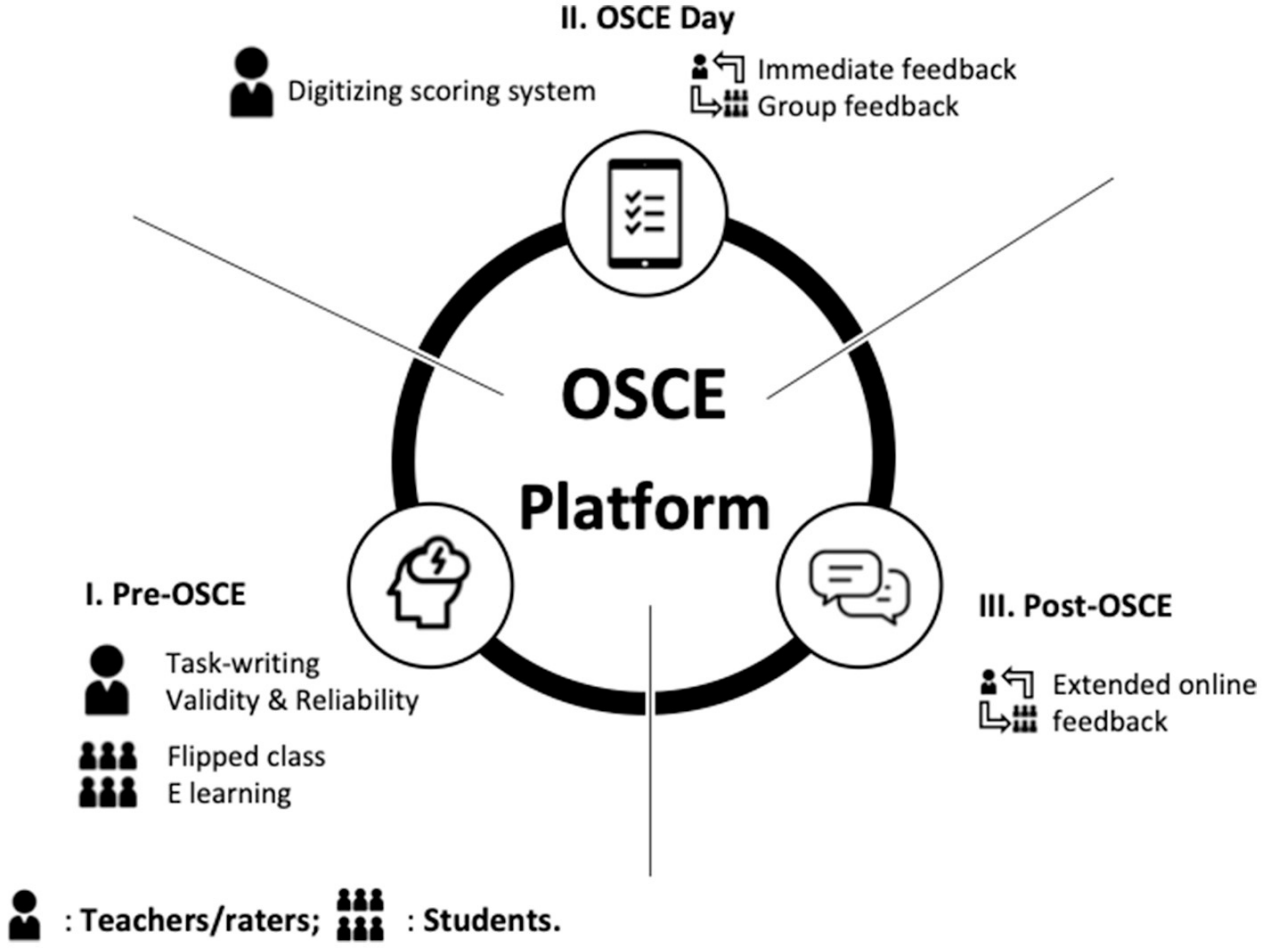
Diagrammatic framework of Online Smart Communicative Education System. The online system comprises (I) Pre-objective structured clinical examination (OSCE): an online flipped class for preparation of task-related knowledge and skills for students, and task-writing for submission to teachers; (II) OSCE-day: 10 tasks in one track formative OSCE and a digitizing scoring system with educational support for teachers with immediate and group feedback; and (III) Post-OSCE: extended online feedback for participants with further questions after the exam and opportunities for teachers to provide more feedback following the review of their performance online.

**Figure 2:**
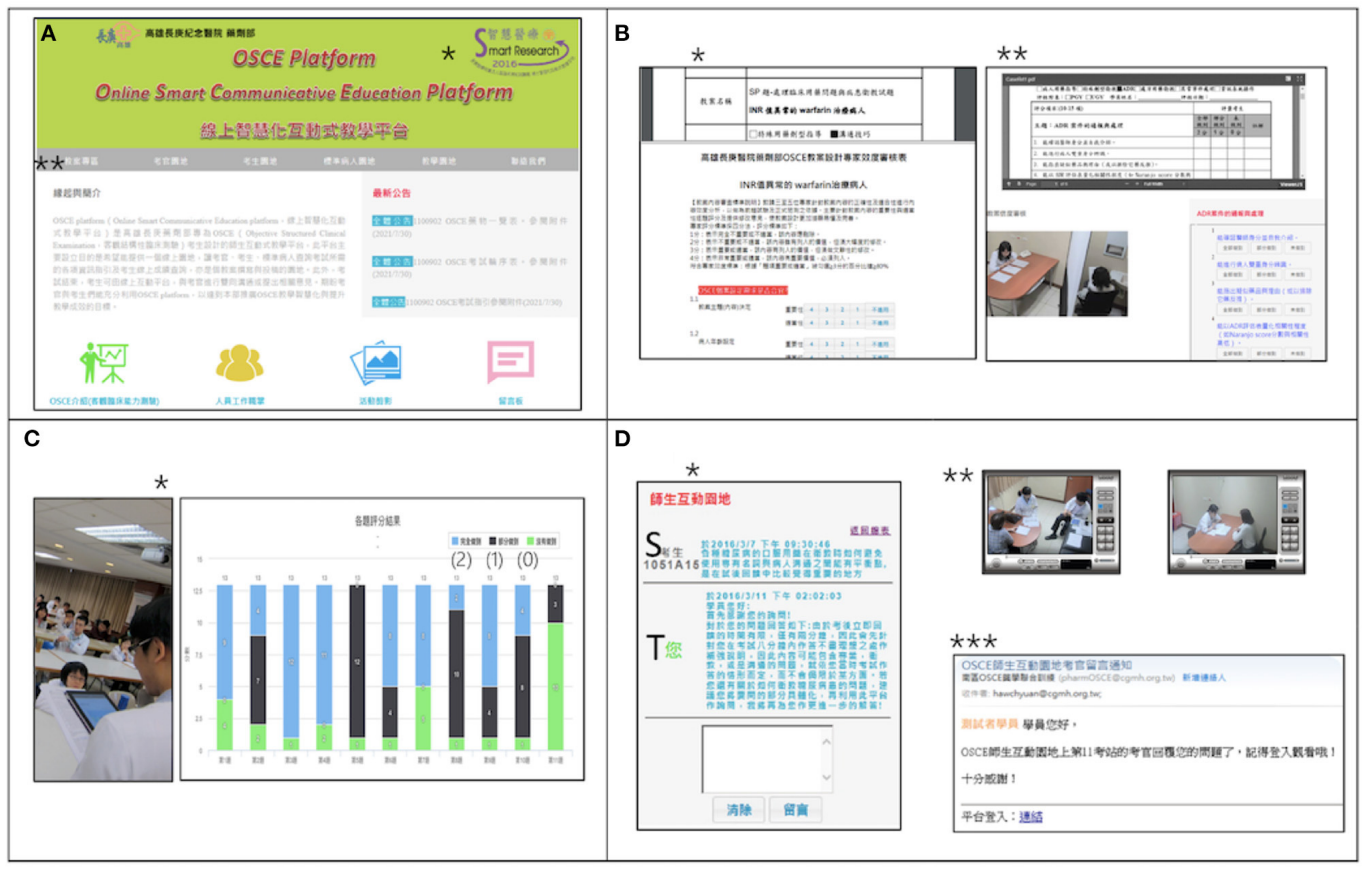
Online Smart Communicative Education System. **(A)** Homepage of Online Smart Communicative Education System (https://www1.cgmh.org.tw/intr/intr4/c8g000/OSCE/OSCE_index.asp). *Smart Research Award of Smart Hospital by Joint Commission of Taiwan in 2016; **The icons indicate the entries of “Task-submission,” “Raters,” “Students,” “Standardized patients,” “Online teaching forum,” and “Contact.” **(B)** Task submission: It provides task-submission, peer-review, online video, and checklist for analysis of validity (*) and reliability (**simultaneous online video with a rating for reliability). **(C)** Circuit feedback: The program director debriefs common mistakes from automatic plotting and real time calculation by mobile devices (*) instantly; “(2), (1), (0)”: the scale for rating; bars with different colors depict participants' responses). **(D)** Extended online feedback: *Students (S) may access to ask more questions after OSCE and teachers (T) can provide more feedback; **online video will be available for teachers to review students' performance; ***simultaneous e-mail notification once the students drop the questions to teachers or the teachers' response to students.

We provided an integrated online communicative program to help students' learning and to facilitate the scoring and feedback for the teachers. The aim of the program is to support teachers/raters in submitting tasks, undertake online inter-rater and intra-rater reliability analyses, and provide extended online feedback. For the students/learners, we provided a pre-OSCE preparatory online flipped class comprising related knowledge and skills for students from different training hospitals, a pre-OSCE online test, and extended online feedback. Students were required to pass the pre-OSCE online test prior to being approved to participate in the OSCEs. The mobile scoring system was utilized for the raters' training to achieve rating consensus and students' scoring during the OSCEs, thereby enabling a real-time, statistical, and automated calculation, while plotting for immediate and group feedback. To achieve this, we developed an Online Smart Communicative Education System comprising an online communicative forum and a digitizing scoring system (Figures 1, 2). The Internet communicative forum included a task-submission with peer review to reach consensus and to provide extended online feedback to the learners. The digitizing scoring system was developed based on mobile devices connected by wireless fidelity (Wi-Fi) with real-time scoring, automatic plotting, and calculation.

### Constructive Feedback Model

We designed an extended online feedback forum in addition to the immediate one after each task, and group response for all students in every circuit at the end of their OSCEs. Participants could ask further questions online during the week following the exam and the raters could review their video online and provide more specific feedback individually.

### Questionnaires for the Perceptions of Students and Raters of the Online Smart Communicative Education System

We developed two questionnaires to investigate the perceptions of the participants of the Online Smart Communicative Education System. The students' questionnaire explores factors that potentially affect the process of learning. The raters' questionnaire investigates factors that potentially affect the process of teaching with educational support. To ensure the content validity of the instrument, the items of the instrument were developed by experienced clinical teachers based on the feedback from the students and raters in previous OSCEs. The structures of the instruments for the students were flipped class and feedback, and those for the teachers were rating-consensus, educational support of digitizing scoring system, and feedback. Four senior clinical pharmacists and one educator were invited to examine the contents for expert validity. Ten items for the students and thirteen items for the raters to examine the processes of teaching and learning were developed. The reliability was tested by Cronbach's alpha. The online questionnaires were open after the OSCE online and closed 2 weeks after the OSCE.

### Statistics and Analysis

Quality assurance was analyzed *via* a generalizability study with EDU-G software, with a Decision-study being applied to optimize the minimal number of tasks (an expected G value > 0.8). The satisfactory agreements were graded using a Likert scale (1–5 points from complete disagreement to complete agreement). Principal component analysis (PCA) with varimax rotation was performed to analyze the factors affecting the program as measured by the questionnaire. Significance was set at *P* < 0.05.

## Results

Seventy-six students (36 UGY and 40 PGY; male, 32.9%) and 24 raters (male, 25%) participated. The mean scores of the checklist were 749.25 ± 64.6. The overall satisfactory agreements of the program for the students were as follows: pre-OSCE flipped class 4.06 ± 0.72, OSCE-day orientation and introduction 4.24 ± 0.66, and facilitation of learning 4.61 ± 0.52. The self-evaluation of core competencies assessed by the students themselves improved significantly before and after the program (*P* < 0.001).

### Quality Assurance Analysis by Generalization Study

The generalizability study showed the variances of participants 32.8, 55.6, 33.4, and 50.4%, respectively, on different days. The differentiation variances of participants were 66.3, 99.5, 61.3, and 113.6%, respectively, with an absolute error variance for participant-raters: 13.605 (100%), 7.445 (93.8%), 12.216 (100%) and 11.197 (100%). The mean relative/absolute coefficient G value was 0.876/0.875 (0.830–0.930/0.830–0.926) (Table 1). For the Decision study, we fixed the participants, then optimized them by adjusting the numbers from eight and nine to 11 and 12 tasks. This resulted in the expected G value of 0.814 with a minimum of nine tasks in the cohort (Table 2).

**Table 1:** Generalizability ANOVA.

**Year**	**D**	***n***		**Variance (%)**			**DV**	**Absolute error variance (%)**		**Coef_G**	
		**P**	**R**	**P**	**R**	**PR**	**P**	**R**	**PR**	**Rel**.	**Abs**.
2016	1	19	10	32.8	0	67.2	66.266	–	13.605 (100%)	0.83	0.83
	2	20	10	55.6	2.7	41.6	99.450	0.488 (6.2%)	7.445 (93.8%)	0.930	0.926
2017	3	15	10	33.4	0	66.6	61.286	–	12.216 (100%)	0.834	0.834
	4	22	10	50.4	0	49.6	113.584	–	11.197 (100%)	0.910	0.910
**Total**		**76**	**40**								
**Mean**										**0.876**	**0.875**

*Abs, absolute; Coef_G, coefficient G; D, Day; DV, differentiation variance; P, participant; SD, standard deviation; R, rater; Rel, relative*.

**Table 2:** Optimization of decision study by task numbers.

			**G-study**		**Option 1**		**Option 2**		**Option 3**		**Option 4**	
			**Lev**.	**Univ**.	**Lev**.	**Univ**.	**Lev**.	**Univ**.	**Lev**.	**Univ**.	**Lev**.	**Univ**.
		P	19	INF	19	INF	19	INF	19	INF	19	INF
		D	1	INF	1	INF	1	INF	1	INF	1	INF
	D1	T	10	INF	8	INF	9	INF	11	INF	12	INF
		Observ.	190		152		171		209		228	
		G Rel.	0.83		0.796		0.814		0.843		0.854	
2016		G Abs.	0.83	0.796	0.814	0.843	0.854
		P	20	INF	20	INF	20	INF	20	INF	20	INF
		D	1	INF	1	INF	1	INF	1	INF	1	INF
	D2	T	10	INF	8	INF	9	INF	11	INF	12	INF
		Observ.	200		160		180		220		240	
		G Rel.	0.93		0.914		0.923		0.936		0.941	
		G Abs.	0.926		0.909		0.919		0.932		0.938	
		P	15	INF	15	INF	15	INF	15	INF	15	INF
		D	1	INF	1	INF	1	INF	1	INF	1	INF
	D3	T	10	INF	8	INF	9	INF	11	INF	12	INF
		Observ.	150		120		135		165		180	
		G Rel.	0.834		0.801		0.819		0.847		0.858	
2017		G Abs.	0.834		0.801		0.819		0.847		0.858	
		P	22	INF	22	INF	22	INF	22	INF	22	INF
		D	1	INF	1	INF	1	INF	1	INF	1	INF
	D4	T	10	INF	8	INF	9	INF	11	INF	12	INF
		Observ.	220		176		198		242		264	
		G Rel.	0.91		0.89		0.901		0.918		0.924	
		G Abs.	0.91		0.89		0.901		0.918		0.924	

*Abs, absolute; G, coefficient G; D, Day; DV, differentiation variance; INF, infinite; Lev., level; Observ., observation; P, participant; SD, standard deviation; T, task; Rel, relative; Univ, universe*.

### Perceptions of the Students to the Online Smart Communicative Education System

Seventy-six questionnaires were obtained from the students for the analysis (response rate 100%). The Cronbach's alpha of the questionnaires was 0.878. Ten items could be categorized into a two-component model with eigenvalues > 1 (6.744 and 2.123) (Table 3). Principal component analysis (PCA) with varimax rotation showed a Kaiser-Meyer-Olkin (KMO) value of 0.822 with a significant Bartlett sphericity test (*P* < 0.001). It could explain the cumulative variance of 73.9% in *component (1) “Effects of extended online feedback*”: 40.017%, and *component (2) “Facilitation of learning”*: 33.9%, respectively.

**Table 3:** Factor analysis affecting the students' perceptions.

		**Factor extraction**		
		**Initial**	**Component**	
	**Mean ±SD**		**1**	**2**
1. Online flipped class may help me prepare OSCEs.	4.06 ± 0.722	0.646	0.3	0.745
2. Introduction on OSCE day may help me understand how to do it.	4.24 ± 0.664	0.74	0.32	0.799
3. Time is limited for me to ask questions during immediate feedback.	3.65 ± 1.717	0.794	0.85	0.265
4. I feel free to ask questions online.	3.72 ± 1.617	0.91	0.91	0.285
5. I may ask more questions online after OSCE.	3.41 ± 1.871	0.763	0.834	0.261
6. OSCEs help me to know my own competencies.	3.77 ± 1.377	0.702	0.754	0.365
7. I can get useful feedback from raters.	3.34 ± 1.907	0.748	0.778	0.378
8. Feedback is critical for my learning.	3.68 ± 1.622	0.872	0.863	0.357
9. Online platform is feasible for me.	4.34 ± 0.597	0.828	0.174	0.893
10. Online platform is useful for my learning.	4.23 ± 0.750	0.843	0.076	0.915
% Of variance			40.017	33.874
Cumulative % of variance				73.891

*SD, standard deviation*.

The items of “I feel free to ask questions online (0.91),” “Feedback is critical for my learning (0.86),” “Time is limited for me to ask questions during immediate feedback (0.85),” and “I may ask questions online after OSCE (0.83)” are the major factors in *component 1*. The items of “Online platform is useful for my learning (0.92),” “Online platform is feasible for me (0.89),” “Introduction on OSCE day may help me understand how to do it (0.8),” and “Online flipped class may help me prepare OSCE (0.75)” are the major factors in *component 2*. The mean expected length of response from the teachers was 7.87 ± 5.75 days.

### Perceptions of the Raters to the Online Smart Communicative Education System

Forty questionnaires were sent to the teachers who had experienced the Online Smart Communicative Education System and 39 questionnaires were obtained for the analysis (response rate 97.5%; males, 23.1%). The Cronbach's alpha of the questionnaires was 0.920. The PCA with varimax rotation showed a KMO value of 0.780 with a significant Bartlett sphericity test (*P* < 0.001). Thirteen items could be categorized into three groups as a three-component model with eigenvalues >1 (ranging from 1.323 to 5.62) (Table 4). The three-component model includes the following *component (1) Effects of extended online feedback, (2) Facilitate scoring and feedback*, and (*3) Feasibility of online platform*.

**Table 4:** Factor analysis affecting the teachers' perceptions.

		**Component**		
	**Initial**	**1**	**2**	**3**
1. Online platform may help me to achieve consensus more efficiently with other raters.	0.642	0.033	0.8	0.035
2. DSS helps me avoid missing items.	0.576	0.042	0.751	0.096
3. Real time calculation helps me for immediate feedback.	0.775	–	0.867	0.07
4. DSS helps me make remarks.	0.503	–	0.486	0.475
5. DSS is useful for group feedback.	0.708	–	0.771	0.31
6. DSS helps my rating more efficiently.	0.403	–	0.54	0.274
7. Extended online feedback helps me provide more feedback for students.	0.864	0.925	–	–
8. Extended online feedback may help students to ask more questions online.	0.983	0.974	–	–
9. Extended online feedback helps provide more opportunities for shy students to ask.	0.965	0.925	–	–
10. Extended online feedback helps me provide much complete feedback to students.	0.95	0.968	–	–
11. Extended online feedback may help my teaching.	0.971	0.968	–	–
12. Online platform is convenient for me.	0.855	–	0.19	0.898
13. Online platform is useful for me.	0.854	–	0.182	0.899
% of Variance		36.619	24.475	16.199
Cumulative % of Variance				77.294

*DSS, digitizing scoring system*.

The major factors of *component (1) Effects of extended online feedback* comprised “Extended online feedback may help students to ask more questions online (0.97),” “Extended online feedback helps me provide much complete feedback to students (0.97),” “Extended online feedback may help my teaching (0.97),” “Extended online feedback helps me provide more feedback for students (0.92),” and “Extended online feedback helps provide more opportunities for shy students to ask (0.93).” The major factors of *component (2) Facilitate scoring and feedback* comprised “Real time calculation helps me for immediate feedback (0.87),” “Online platform may help me to achieve consensus more efficiently with other raters (0.80),” “Digitizing scoring system (DSS) is useful for group feedback (0.77),” “DSS helps me avoid missing items (0.75),” “DSS helps my rating more efficiently (0.54),” and “DSS helps me make remarks (0.486).” *Component (3) Feasibility of online platform* comprised “Online platform is useful for me (0.899)” and “Online platform is convenient for me (0.898).” The explained cumulative variance was 77.3% in *components (1) “Effects of extended online feedback”: 36.6%, (2) “Facilitation of scoring and feedback”: 24.5%*, and (*3) “Feasibility of online platform”: 16.2%*, respectively.

## Discussion

We established and integrated an online platform including (i) a flipped class for students to prepare themselves prior to the traditional formative OSCE, (ii) a mobile scoring system, to facilitate teachers' rating and to avoid unintentionally missing the items, with benchmark a more convenient manner for immediate feedback, (iii) real time automatic calculation and plotting for debriefing common mistakes in groups, and (iv) an extended online forum for the students, enabling them to ask more questions following reflection and to enable teachers to provide more feedback. Our findings build on the work undertaken by Kropmans et al. (9) and Meskell et al. (10), who explored assessors' perceptions and benefits based on an electronic OSCE management system. They concluded that the high assessors' satisfaction and analysis of the assessment results could highlight issues of internal consistency. Moving the field forward, our study ascertained good reliability of mobile device scoring. Using a generalizability study, we identified students as the major source of variance, with much less variation from the raters. We extrapolated a minimum number of tasks to achieve our expected G coefficient using a Decision-study, which investigated the possibility of increasing the length of feedback by decreasing task numbers whilst maintaining good reliability. However, we were unable to decrease task numbers and increase feedback length because our blueprint was designed prior to the exam.

Factor analysis of students' and raters' perceptions found that the “Effects of extended online feedback” comprised the most important part of the process of learning and teaching. “I feel free to ask questions online” was the highest factor in students' perceptions. One of the possible reasons might be that students are used to communicating or interacting with others *via* smartphones or other electronic devices. For raters, they believed that having questions in an online forum encouraged shy students to engage better. Furthermore, teachers expressed a desire to offer better and complete feedback to students online.

Using mobile devices for scoring was considered to be convenient for the raters to make better observations with simultaneous scoring. It could explain the second important part of variance for the teachers. The utilization of mobile devices with real time statistics, user-friendly scoring, and benchmarks would provide much educational support for the teachers in comparison with traditional formative OSCEs. Our study demonstrated the feasibility and security of the online digitizing process to manage big data of complicated multi-task information from learners and raters.

The OSCEs are commonly used to assess desired competencies in healthcare students. However, limited time causes stress among students, resulting in unanswered questions for students and haste among teachers when scoring and providing feedback. However, formative feedback is considered important for the provision of critical information in response to learners' performance including verification of response accuracy, explanation of the correct answer, hints, and worked examples (20). Such feedback can be immediately administered following an answer, or after some time has elapsed to achieve and to promote learning. We have constructed a framework of feedback including (i) immediate feedback, whereby all students are provided with individual and timely feedback from the raters, immediately following each task; (ii) Circuit feedback, whereby the director presents common mistakes drawing on automatic plotting and real time calculation by mobile devices, with students engaged in peer discussion following each circuit; (iii) Extended online feedback, with extended, dynamic teaching, and learning processes occurring online following the OSCE; and (iv) Individual written feedback, based on blueprints, provided to every student following their online feedback. To our knowledge, extended online feedback is a novel approach, extending students' reflection and the feedback time to facilitate learning and teaching within a formative OSCE.

### Strength and Limitations of the Study

All research has limitations. Although our study included 14 hospitals with varying capacities, all of these were in the southern part of a single country. Further studies are needed to ascertain the degree to which this type of scoring system is internationally acceptable alongside accessibility and reliability, web speed, and security concerns. Another limitation is the relatively small sample size in conducting our analysis: *n* = 76 for students and *n* = 39 for raters. While we believe that our student data is relatively robust, we are concerned with the reliability of the rater results. However, we do note that, in the world of factor analysis, guidelines have been called into question for their conservative nature, with researchers obtaining good results with relatively small sample sizes (21). We, therefore, urge caution around the interpretation of our data due to low-rater numbers, although we feel that the results are worthy of being reported. Despite the limitations, our study has strengths. For example, we have established a network and teamwork for teachers and students across many hospitals and provided a more satisfactory way of assessing and educating students.

### Future Research

As technology progresses in terms of hardware or software, we may integrate more advanced facilities to improve our learning and teaching, especially during the COVID-19 pandemic. Although our study addressed some issues related to formative OSCEs, more advanced research, considering the optimal length of the feedback in formative OSCEs and the impact of constructive critical feedback for teachers and students, should be validated and investigated.

## Conclusion

Our study demonstrated a novel approach toward extended online feedback to aid and augment the learning of health professionals successfully. We demonstrated good reliability for digitizing a scoring system with educational support to facilitate the teaching of the raters. This interactive online platform provides a forum and extends the process of learning and teaching, thereby overcoming the barriers of time limitations and distance as compared with traditional formative OSCEs.

## Data Availability Statement

The original contributions presented in the study are included in the article/supplementary material, further inquiries can be directed to the corresponding author.

## Ethics Statement

The studies involving human participants were reviewed and approved by Institutional Review Board Ethical Committee of Chang Gung Medical Foundation. The participants provided their written informed consent to participate in this study.

## Author Contributions

The final version was approved for publication by all authors and agreed to be accountable for all aspects of the work. All authors have contributed to this paper to the design of the study, developing the questionnaires, the acquisition of data for the work, and draft writing.

## Funding

This work was granted by Kaohsiung Chang Gung Memorial Hospital, Chang Gung Medical Foundation, Taiwan, Republic of China (Grant Numbers: CDRPG 8H0061-62, 8J0021-22, and 8L0021).

## Conflict of Interest

The authors declare that the research was conducted in the absence of any commercial or financial relationships that could be construed as a potential conflict of interest.

## Publisher's Note

All claims expressed in this article are solely those of the authors and do not necessarily represent those of their affiliated organizations, or those of the publisher, the editors and the reviewers. Any product that may be evaluated in this article, or claim that may be made by its manufacturer, is not guaranteed or endorsed by the publisher.
